# Difficulties in emotion regulation and exercise addiction among retired adults: the mediating roles of state anxiety and social dependence

**DOI:** 10.3389/fpsyg.2026.1752353

**Published:** 2026-02-05

**Authors:** Dan Huang, Hanyue Lei, Tang Chao, Denise Koh

**Affiliations:** 1 School of Physical Education, Hunan University of Science and Technology, Xiangtan, China; 2 Faculty of Education, Universiti Kebangsaan Malaysia, Bangi, Malaysia; 3 Sport Science School, Beijing Sport University, Beijing, China

**Keywords:** emotion regulation, exercise addiction, retired adults, social dependence, state anxiety

## Abstract

**Introduction:**

Exercise is often viewed as universally beneficial for older adults; however, a growing body of evidence suggests that excessive or compulsive exercise may be linked to underlying psychological vulnerabilities. In later life, retirement-related role changes and emotional challenges may increase reliance on exercise as a means of emotion regulation, potentially elevating the risk of maladaptive exercise patterns. Against this backdrop, the present study focuses on retired older adults who engage in regular exercise, examining the psychological mechanisms underlying exercise addiction.

**Methods:**

This study investigates the role of difficulties in emotion regulation in predicting exercise addiction among retired individuals, with state anxiety and social dependence as mediators. Data were collected from 471 retired Chinese older adults with regular exercise habits through on-site paper-based surveys. Participants reported engaging in regular exercise, defined as planned physical activity performed at least three times per week for health or recreational purposes. The participants had an estimated mean age of 60.76 years (SD = 8.31). The sample consisted of 144 males (30.6%) and 327 females (69.4%). Validated scales were used to assess difficulties in emotion regulation, state anxiety, social dependence, and exercise addiction. Partial least squares structural equation modeling (PLS-SEM) was conducted to test the hypothesized model and examine both direct and indirect associations.

**Results:**

Difficulties in emotion regulation were positively associated with state anxiety (*β* = 0.811, *p* < 0.001) and social dependence (*β* = 0.327, *p* < 0.001). Both state anxiety (*β* = 0.312, *p* < 0.001) and social dependence (*β* = 0.332, *p* < 0.001) were positively associated with exercise addiction, and each served as a significant mediator linking emotion regulation difficulties to exercise addiction.

**Conclusion:**

Exercise addiction among older adults is shaped by both emotional and social vulnerabilities. Interventions should therefore focus on improving emotion regulation abilities and strengthening social support to reduce the risk of compulsive exercise.

## 1 Introduction

Chinese society is experiencing a rapid demographic shift toward aging, between 2020 and 2050, the number of individuals in China aged 65 years and older is projected to increase from approximately 172 million, accounting for 12 percent of the total population, to around 366 million, representing 26 percent (Feng et al., 2020). This trend has resulted in a substantial increase in the size of the post-retirement population, generally referring to adults aged 60 years and above, a group that typically overlaps with the middle-aged and older adult demographic. As people exit the workforce and transition into retirement, they often undergo significant changes in their social roles, daily routines, and sources of identity and support (Bordia et al., 2020). These shifts, coupled with the challenges of aging, have intensified the psychological burden faced by post-retirement individuals. Common mental health issues in this group include depression, anxiety, cognitive decline, and loneliness (Dave et al., 2008), all of which are closely associated with impaired emotion regulation capacities and increased difficulties in managing negative affect. Empirical studies have reported that approximately 15.7% of older men and 22.2% of older women in China exhibit depressive symptoms (Li et al., 2014), with the higher prevalence among women often attributed to greater caregiving burdens, lower socioeconomic resources, and heightened emotional sensitivity in later life, indicating a notable mental health burden among post-retirement individuals. These concerns are often driven by role loss following retirement, increased physical vulnerability due to chronic illness (Awang et al., 2018), reduced family companionship (Bosma et al., 2025), and shrinking social networks (Ayalon, 2019). Moreover, many post-retirement individuals experience a diminished sense of control over their future, especially regarding fears of disability, cognitive impairment, or becoming a burden to their families—factors that may exacerbate emotional distress (Jensen et al., 2020). These interconnected challenges significantly impact their quality of life and psychological well-being, highlighting the urgent need for targeted public health responses and mental health services tailored to this growing population segment.

In addition to well-documented mental health concerns such as depression, anxiety, and loneliness, certain psychological risks among older adults remain relatively hidden yet equally worthy of attention (Kaimara et al., 2022). Some of these risks are masked by their outward appearance as healthy behaviors and are often overlooked or misinterpreted as desirable lifestyle habits. One such example is exercise addiction, a mental health risk masked by the widely held belief that physical activity is inherently beneficial (Hausenblas et al., 2017). Within the Chinese context, the development of exercise addiction among older adults is driven by multiple interrelated factors. Health-related anxiety is a prominent motivator (Berczik et al., 2012). As individuals age and encounter increasing health concerns or chronic conditions, some become highly sensitive to changes in their physical status (Helgeson and Zajdel, 2017). They may come to view exercise not only as a health-promoting activity but also as a central means of disease management and aging prevention, which may foster psychological dependence over time (Back et al., 2021). When exercise routines are disrupted, these individuals may experience heightened anxiety, guilt, or emotional discomfort (Mason et al., 2019). Changes in social roles following retirement also contribute significantly. Many older adults lose their occupational identity and channels for formal social participation, leading to a profound restructuring of daily life (Goll et al., 2015). In the absence of new goals or structured routines, regular exercise is often imbued with new functional meaning—it not only serves to maintain physical health but also becomes a means of restoring daily order and re-establishing a sense of purpose (Mobily et al., 2017). In some cases, individuals begin to equate persistent physical activity with personal worth or self-discipline, progressively increasing their exercise frequency and intensity (Mappanasingam et al., 2024). Moreover, exercise often serves an important social function among older adults, potentially fostering social dependence through repeated social interaction. Activities such as group dancing, walking clubs, and morning exercise routines provide not only physical stimulation but also opportunities for social connection and emotional support (Stathi et al., 2002). For those who lack family companionship or have limited social resources (Hirsch and Clark, 2019), exercise may become a primary channel for fulfilling needs for belonging and interpersonal affirmation (Mack et al., 2017). In such cases, exercise shifts from a physical necessity to an emotional and social coping mechanism. Additionally, a lack of diverse emotion regulation strategies can reinforce the risk of psychological dependence (Aldao et al., 2015). Some older adults have limited tools to manage stress, loneliness, or emotional discomfort, and may rely exclusively on physical activity to modulate their internal states (Kwag et al., 2011). Over time, this over-reliance can lead to compulsive patterns of exercise behavior (Hausenblas and Symons Downs, 2002). It is also important to note that, compared to younger individuals, older adults are generally less aware of the potential negative consequences of excessive exercise (McPhee et al., 2016). Social and familial environments often interpret frequent physical activity as a sign of vitality and self-discipline, inadvertently neglecting the underlying psychological drivers (Lichtenstein et al., 2014). This common misperception obscures the nature of the issue and makes identification and intervention more challenging.

Once older adults develop exercise addiction, they may face a range of physiological and psychological risks, including stress fractures, joint damage, and heightened anxiety (Lichtenstein et al., 2017). Physiologically, excessive exercise can exacerbate joint damage and trigger cardiovascular events, posing particular dangers for older adults with chronic illnesses or osteoporosis (Watso and Vondrasek, 2024). Psychologically, individuals with exercise addiction often develop a dependence on physical activity; interruptions can provoke anxiety and mood fluctuations (Freimuth et al., 2011), while increasing neglect of family and social relationships, ultimately leading to emotional isolation. At the lifestyle level, overinvestment in exercise displaces other essential activities, disrupts life balance, and may impair adaptive capacity due to the compulsion to maintain uninterrupted exercise (Swainson and Philip-Rafferty, 2018). Furthermore, an excessive belief equating exercise with health often leads to ignoring bodily signals, resisting rest or medical advice, and even interfering with the proper management of chronic conditions (Ekkekakis et al., 2016). These factors interact, creating a covert yet serious threat to the health of older adults (Corner and Bond, 2004). Given these complexities, elucidating the mechanisms underlying exercise addiction in older adults is a critical prerequisite for understanding and effectively intervening in this phenomenon (Rejeski and Fanning, 2019).

Exercise addiction should not be regarded merely as a physiological or behavioral issue; rather, it emerges from the interplay of multiple psychological drivers, social contexts, and individual cognitions (Egorov and Szabo, 2013). A lack of systematic knowledge about its developmental pathways limits societal and familial recognition and response and constrains the evidence base for policy interventions and individualized guidance (Brady et al., 2020). Therefore, clarifying the trajectory by which older adults’ exercise behaviors shift from healthy engagement to compulsive dependence, and identifying key psychological drivers and moderating factors, is essential for establishing more rational exercise participation paradigms and for developing precise intervention strategies (Clark et al., 2019). Research in this area not only addresses pressing needs in the field of geriatric mental health but also extends our understanding of addictive behaviors in nontraditional populations.

Although physical activity is widely recognized as a crucial means of enhancing the physical and mental health of older adults, existing research has predominantly focused on the benefits of moderate exercise, such as improved cognitive function (Tse et al., 2015), alleviation of depressive symptoms (Blake et al., 2009), and enhanced physical capacity (Hogan, 2005). However, as the frequency and intensity of exercise among older adults increase, some studies have begun to acknowledge the potential “double-edged sword” effect of exercise—where excessive or compulsive exercise, involving rigid and difficult-to-control exercise patterns, may lead to physiological harm (Nogueira et al., 2019), including joint wear and cardiovascular risks—yet the underlying psychological mechanisms and behavioral patterns remain underexplored. In particular, under the prevailing belief that “exercise equals health,” exercise addiction remains an overlooked but potentially significant psychological concern among older adults (Godoy-Izquierdo et al., 2023). Current literature is largely descriptive, lacking systematic theoretical frameworks and empirical validation, with research on underlying mechanisms such as addictive motivation, emotion regulation, and social dependence extremely limited (Egorov and Szabo, 2013). Furthermore, standardized criteria for identifying exercise addiction in older adults, validated measurement instruments, and effective intervention strategies have yet to be fully developed (Falck et al., 2016).

To address this research gap, the present study investigates the psychological mechanisms underlying exercise addiction in retired adults by constructing a path model that incorporates difficulties in emotion regulation, state anxiety, and social dependence. Specifically, it examines whether difficulties in emotion regulation indirectly contribute to exercise addiction through heightened state anxiety, whether social dependence mediates the link between emotion regulation difficulties and compulsive exercise behaviors, and whether state anxiety and social dependence exert independent or joint mediating effects. By clarifying these pathways, the study provides theoretical grounding and empirical evidence for understanding and intervening in exercise addiction within retired populations.

## 2 Literature review and hypothesis development

### 2.1 Concept of variables

#### 2.1.1 Difficulties in emotion regulation

Difficulties in emotion regulation refer to the obstacles or challenges individuals encounter in identifying, understanding, accepting, or modulating their emotions, reflecting weaknesses in emotional management rather than the intensity or frequency of emotions themselves (Gratz and Roemer, 2004). The concept was systematically proposed by Gratz and Roemer (2004), and has since evolved from an initial focus on specific regulation strategies to a multidimensional model addressing individual differences and psychological health. Over time, this construct has been widely applied across diverse cultural contexts and in both clinical and non-clinical populations, consistently demonstrating strong relevance to psychological well-being (Garnefski et al., 2002).

Difficulties in emotion regulation have been widely applied in various research domains. In clinical psychology, it has been used to explain conditions such as depression (Joormann and Gotlib, 2010), anxiety (Helbig-Lang et al., 2015), borderline personality disorder (Kuo et al., 2015), eating disorders (Ruscitti et al., 2016), and substance abuse (Stellern et al., 2023). In exercise psychology and behavioral addiction studies, difficulties in emotion regulation has been employed to investigate tendencies such as exercise addiction and internet addiction (Hausenblas et al., 2017), with findings indicating that individuals with higher difficulties in emotion regulation are more prone to compulsive exercise behaviors, loss of control over training duration or intensity, and heightened anxiety and mood fluctuations when exercise is restricted. These behaviors not only disrupt daily life balance but may also increase the risk of physical injury. Additionally, difficulties in emotion regulation is widely used as a key outcome measure in intervention studies (Hallion et al., 2018), including mindfulness training (Roemer et al., 2015) and cognitive-behavioral therapy (Berking et al., 2013), to evaluate the effectiveness of such programs. Numerous representative studies have consistently demonstrated a strong association between difficulties in emotion regulation and psychological health problems.

#### 2.1.2 State anxiety

State anxiety refers to a temporary, subjective emotional state characterized by tension, uneasiness, or worry experienced by individuals in specific situations, typically accompanied by physiological arousal and heightened psychological alertness (Marteau and Bekker, 1992). It represents an immediate response to a particular threat or stressor (Prem et al., 2016). Unlike trait anxiety or generalized anxiety, state anxiety is situationally dependent, transient, and fluctuating, varying with changes in the environment or stress context (Endler and Kocovski, 2001).

State anxiety has been widely examined in sports psychology (Ziegler et al., 2009), health psychology, educational psychology (Buckley et al., 2016), and behavioral addiction research (Miller, 2012), reflecting its broad applicability across fields. Among middle-aged and older adults, age-related declines in physiological function and increasing health uncertainty render them more susceptible to state anxiety when facing health examinations, exercise routines, or daily life stressors, and such immediate emotional responses may further influence behavioral choices and lifestyle habits (Mutz et al., 2022).

Compared to generalized or trait anxiety, the advantage of state anxiety lies in its capacity to capture dynamic emotional changes and precisely measure an individual’s psychological response in a specific context, thereby elucidating the real-time interactions among emotional, cognitive, behavioral, and physiological processes (Vigneau and Cormier, 2008). Moreover, it serves as a sensitive short-term indicator for interventions and experimental outcomes, allowing psychological research to move beyond abstract long-term tendencies toward measurable, trackable, and modifiable immediate psychological states, enhancing both precision and practical applicability (Schmidt et al., 2014).

#### 2.1.3 Social dependence

Social dependence refers to an individual’s tendency to rely on others or social relationships for emotional, cognitive, or behavioral support, manifested as seeking emotional reassurance, social recognition, or functional assistance through interpersonal interactions (Hirschfeld et al., 1977). It encompasses both emotional dependence and behavioral dependence (Bornstein, 1992). Social dependence reflects the psychological need for security (Bornstein, 1992), comfort, and companionship (Feeney and Collins, 2015), and may also appear as reliance on others for guidance or participation in decision-making and daily activities (Van Petegem et al., 2012). Among older adults, age-related declines in physiological function, unstable health conditions, and potentially limited social networks often result in higher levels of social dependence (Alonso et al., 2022). This dependence can have protective effects, such as providing emotional support and practical assistance, but it may also contribute to psychological stress or maladaptive behaviors, for instance, excessive reliance on family members or community resources (Kingston et al., 2017).

Previous research has examined social dependence in older adults from perspectives such as attachment theory, social network models, functional ability, and behavioral addiction. For example, Antonucci and Akiyama (1987) indicated through social network and support models that dependent behaviors are influenced by the quality of social support; Chou and Chi (2000) and Berkman et al. (2000) reported that older adults with limited functional abilities rely more on others for daily activities; and Hausenblas et al. (2017) suggested that highly socially dependent older adults may seek emotional connection through exercise or group activities, potentially increasing the risk of overcommitment or addictive behaviors. Overall, social dependence is not only a psychological trait but is also shaped by physiological status, social environment, and functional capacity, making it a crucial variable for understanding psychological well-being, lifestyle, and behavioral patterns in older adults.

#### 2.1.4 Exercise addiction

Exercise addiction refers to a compulsive psychological and behavioral dependence on physical exercise, in which individuals continue to engage in exercise despite negative impacts on their health, daily life, or social relationships, and find it difficult to reduce or stop the behavior (Terry et al., 2004). It differs from regular, healthy exercise, exhibiting typical characteristics of behavioral addiction, including psychological dependence, emotion regulation functions, increased tolerance, neglect of negative consequences, and withdrawal symptoms (Landolfi, 2013).

The notion first emerged in the 1970s, when researchers observed that athletes often persisted in high-intensity training despite fatigue or injury. In the 1980s, the concept of “compulsive exercise” was introduced, linking excessive exercise to psychological dependence. During the 1990s, the construct of exercise addiction became more systematically defined (Di Lodovico et al., 2019), and subsequent research has shown close associations with psychological factors such as perfectionism, difficulties in emotion regulation, state anxiety, and social dependence (Back et al., 2021). More recently, research has expanded beyond athletes to include general and older adult populations, revealing that health anxiety, loneliness, and social dependence may play critical roles in the development of exercise addiction in later life. Methodologically, the field has also diversified, incorporating longitudinal studies, psychological experiments, and intervention-based approaches, thereby deepening understanding of its mechanisms and potential strategies for prevention (Li, 2022). Overall, research on exercise addiction has progressed from behavioral observation to theoretical development, quantitative measurement, and population expansion, providing an important theoretical and empirical foundation for psychology, exercise science, and health interventions.

### 2.2 Hypothesis development

#### 2.2.1 Difficulties in emotion regulation, state anxiety and social dependence

Among retired individuals with regular exercise habits, those with higher difficulties in emotion regulation are more likely to experience State anxiety in response to daily life or exercise-related stressors. Difficulties in emotion regulation reflects deficits in identifying, understanding, accepting, and managing emotions effectively (Faraone et al., 2019). When encountering health issues, unexpected events, or social challenges, these individuals exhibit heightened tension, worry, and physiological arousal (de Groot and van Staveren, 2010). Insufficient emotion regulation amplifies immediate emotional reactivity, increasing the intensity of state anxiety, which can in turn affect exercise behavior, daily routines, and social interactions (Bernstein and McNally, 2018). Thus, difficulties in emotion regulation serves as a psychological vulnerability factor positively influencing State Anxiety, highlighting its central role in the emotional health and behavioral patterns of retired exercisers and providing a foundation for exploring subsequent effects on social dependence and exercise addiction.

Individuals with higher difficulties in emotion regulation often struggle to manage their emotional responses effectively when facing stress or negative effects. As a result, they are more likely to rely on others for emotional support or behavioral guidance (Thoits, 2011). This dependence can manifest as seeking psychological comfort and social recognition, as well as relying on others’ participation or assistance in decision-making and daily activities (Feeney, 2007). In other words, the greater the difficulties in emotion regulation, the more likely individuals are to seek external social resources to maintain emotional stability and psychological security, thereby exhibiting higher levels of social dependence. This relationship is particularly pronounced among retired individuals, who may face declines in physiological functioning, uncertain health conditions, and limited social networks, making them more prone to depend on external support to cope with emotional fluctuations and daily challenges (Thompson et al., 2022). Thus, difficulties in emotion regulation positively predict social dependence, highlighting a key mechanism through which emotion regulation influences social behavior and providing a theoretical foundation for understanding the psychological health, lifestyle, and social patterns of retirees.

Individuals experiencing higher levels of state anxiety often feel immediate tension, unease, and psychological distress, which can prompt them to seek external support to alleviate negative emotions (Leal et al., 2017), thereby exhibiting higher social dependence. Specifically, when facing stressors related to health, daily life, or social situations, heightened State Anxiety increases the need for emotional comfort, social recognition, or behavioral guidance, motivating reliance on family, friends, or broader social networks for psychological support and assistance (Lundqvist et al., 2023). This indicates that state anxiety, as a transient emotional response, positively predicts social dependence; the greater the state anxiety, the more likely individuals are to use social interactions to obtain external resources to mitigate their anxiety. Among retired individuals, who may experience declining physiological functioning and limited social networks, high levels of state anxiety are particularly likely to trigger dependence on social support, influencing daily routines, emotional regulation, and behavioral choices (Kelly et al., 2017). This positive relationship highlights the mechanism through which immediate emotional states shape social behaviors, offering important insights into the psychological health and social adaptation of older adults.

As a result, this study puts forward the following hypotheses:

*Hypothesis* 1 (H1): There is a positive association between difficulty in emotion regulation and state anxiety.

*Hypothesis* 2 (H2): There is a positive association between difficulty in emotion regulation and social dependence.

*Hypothesis* 3 (H3): There is a positive association between state anxiety and social dependence.

#### 2.2.2 State anxiety, social dependence and exercise addiction

State anxiety refers to the transient experience of tension, unease, or worry in response to specific situational stressors (Harrison et al., 2015). When state anxiety levels are elevated, individuals may seek specific behaviors to alleviate or distract themselves from anxiety (Gaspar and McDonald, 2018). Among retired individuals with regular exercise habits, physical activity often serves not only as a health-promoting behavior but also as a means of emotional regulation (Delle Fave et al., 2018). In the presence of high state anxiety, these individuals may be more likely to increase exercise frequency, extend exercise duration, or intensify exercise intensity to reduce psychological tension (Collado-Mateo et al., 2021). While such behaviors may temporarily relieve anxiety, they can gradually develop into Exercise Addiction (Chen, 2016). In this context, state anxiety functions as an immediate negative emotional driver that fosters psychological and behavioral dependence on exercise, making it difficult for individuals to limit or reduce their activity, even when it negatively impacts health, daily life, or social interactions (Rhodes et al., 2017). Thus, a positive relationship exists between state anxiety and exercise addiction, whereby higher levels of state anxiety increase the likelihood of exercise addiction. This mechanism highlights the role of affect-driven behavioral dependence and provides a theoretical basis for understanding exercise behaviors and associated risks in middle-aged and older adults.

Social dependence refers to an individual’s tendency to rely on others or social relationships for emotional, cognitive, or behavioral support (Wang et al., 2015). When individuals exhibit high levels of social dependence, they often engage in specific behaviors to fulfill their need for social and emotional resources (Capone et al., 2020). Among retired individuals with established exercise habits, elevated social dependence may lead them to use physical activity as a primary means of obtaining social contact, emotional support, or a sense of belonging (Lindsay Smith et al., 2017). For instance, they may participate in group exercises, fitness clubs, or community activities to enhance interpersonal interaction and emotional connection (McPhee et al., 2016). Over time, this behavioral pattern may exceed the boundaries of healthy exercise and develop into exercise addiction, characterized by psychological dependence, inability to self-regulate exercise frequency or intensity, and heightened anxiety or emotional fluctuations when exercise is restricted (Godoy-Izquierdo et al., 2023). In this context, higher levels of social dependence increase reliance on exercise as a medium for social and emotional fulfillment, thereby elevating the risk of exercise addiction. This mechanism indicates that social and psychological factors play a critical role in the development and maintenance of exercise behavior in older adults, providing a theoretical basis for understanding the social drivers of exercise addiction.

As a result, this study puts forward the following hypotheses:

*Hypothesis* 4 (H4): There is a positive association between state anxiety and exercise addiction.

*Hypothesis* 5 (H5): There is a positive association between social dependence and exercise addiction.

#### 2.2.3 Mediation effects

Difficulties in emotion regulation reflects individuals’ challenges in monitoring, understanding, accepting, and modulating their emotions (Ritschel et al., 2015). High levels of difficulties in emotion regulation indicate that individuals are less capable of managing negative emotional states effectively, which may lead them to seek external behaviors to cope with psychological distress. Among retired individuals with established exercise habits, difficulties in emotion regulation may indirectly increase the risk of exercise addiction through multiple psychological pathways (Mu et al., 2024). Specifically, individuals with elevated difficulties in emotion regulation are more likely to experience heightened state anxiety in response to situational stressors, which drives them to engage in more frequent, intense, or prolonged exercise as a means of alleviating immediate tension (Hamer and Karageorghis, 2007). Concurrently, difficulties in emotion regulation may increase individuals’ social dependence, prompting them to rely on exercise as a channel for social interaction, emotional support, and a sense of belonging (Dowd et al., 2014). Together, state anxiety and social dependence function as mediators that translate the inability to regulate emotions into maladaptive exercise behaviors, fostering psychological and behavioral dependence on exercise. Therefore, difficulties in emotion regulation can indirectly contribute to exercise addiction via the sequential or parallel mediating effects of state anxiety and social dependence, highlighting the interplay of emotional regulation deficits, transient negative affect, and social motivations in shaping exercise addiction among older adults.

As a result, this study puts forward the following hypothesis:

*Hypothesis* 6 (H6): State anxiety and social dependence mediate the relationship between difficulties in emotion regulation and exercise addiction.

**Figure 1:**
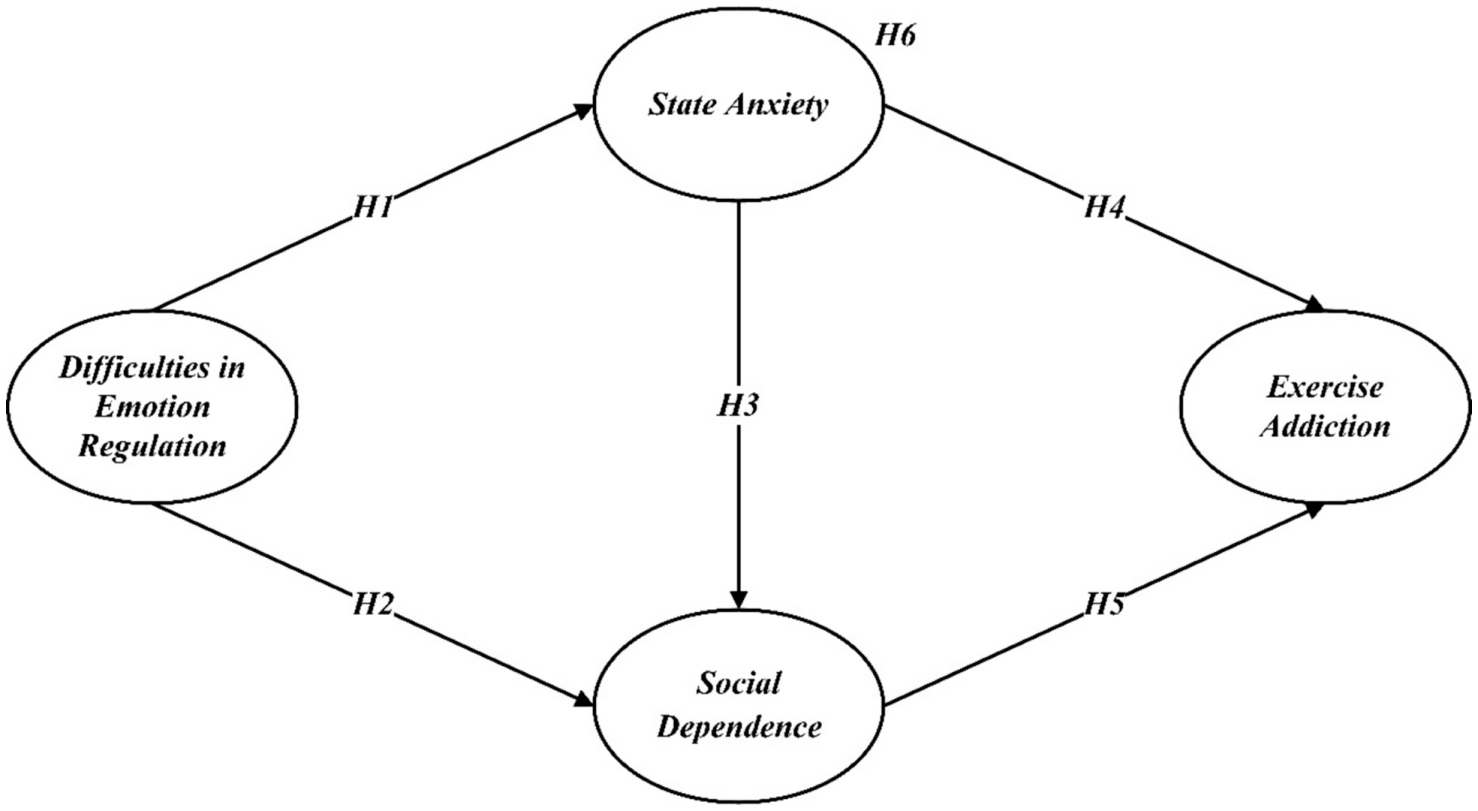
Hypothesized model diagram.

Therefore, the hypothetical model is shown in Figure 1.

## 3 Methodology

### 3.1 Participants and procedures

This study employed a paper-based questionnaire to survey retired adults with regular exercise habits in Hunan Province, China. In the Chinese context, retirement typically begins at 60 years of age for men and at 50 or 55 years of age for women, depending on their occupation and employment type. To capture this target group, on-site data collection was conducted in parks, public fitness squares, and sports centers—common venues where retirees engage in physical activity. With the support of local community organizations and after obtaining verbal informed consent, trained investigators distributed and collected questionnaires face-to-face. A total of 526 questionnaires were distributed, of which 471 were deemed valid after excluding questionnaires with substantial missing data or incomplete responses, yielding a response rate of 89.5%.

Table 1 presents the demographic characteristics of the 471 retired adults in this study. The majority of respondents were female (69.4%), while 30.6% were male. In terms of age distribution, the largest group was aged 50–59 years (60.1%), followed by 17.2% aged 60–69, and 22.7% aged 70 or above. This reflects a relatively young sample within the retirement-age population, with many participants in the early post-retirement stage, a group often examined in studies of older adults. Age was recorded in categories. The mean age and standard deviation were estimated using group midpoints, with the open-ended category (70 years or above) assumed to represent the 70–79 age range. Regarding health status, 29.7% reported at least one chronic medical condition, whereas 70.3% indicated no such conditions. In terms of medication use, 31.0% were on long-term treatment regimens, while the majority (69.0%) reported not relying on ongoing pharmacological therapies. These figures suggest that while a substantial proportion of the sample maintained relatively good health, a considerable number were also managing chronic conditions typical of later life.

**Table 1:** Demographics of sample.

Profiles	Categories	Frequency	Survey (%)
Gender	Male	144	30.6
	Female	327	69.4
Age	50–59	283	60.1
	60–69	81	17.2
	70 or above	107	22.7
Underlying medical conditions	Yes	140	29.7
	No	331	70.3
Use of long-term medications	Yes	146	31.0
	No	325	69.0

### 3.2 Measures

The questionnaire consisted of five sections. The first section collected demographic information, including gender, age, underlying medical conditions and use of long-term medications.

The second section of the survey employed the 8-item Difficulties in Emotion Regulation scale developed by Penner et al. (2023) to assess respondents’ ability to regulate emotions when experiencing distress. Sample items include: “When I’m upset, I have difficulty getting work done”. Responses were rated on a five-point Likert scale ranging from 1 (Strongly Disagree) to 5 (Strongly Agree), with higher scores indicating greater difficulties in emotion regulation.

The third section of the survey employed the 6-item short-form State Anxiety Inventory (STAI-6) developed by Marteau and Bekker (1992) to assess participants’ momentary anxiety levels. Sample items include: “I am tense”. All responses were rated on a five-point Likert scale ranging from 1 (Not at all) to 5 (Very much so), with higher scores reflecting greater levels of state anxiety. Reverse-coded items were rephrased to maintain consistency in direction.

The fourth section of the survey utilized the 6-item Interpersonal Dependency Inventory (IDI-6) developed by McClintock et al. (2015) to measure participants’ reliance on others in emotional and functional domains. The scale includes two subdimensions: emotional dependency (e.g., “I must have one person who is very special to me”) and functional dependency (e.g., “I would rather be a follower than a leader”). Responses were rated on a five-point Likert scale ranging from 1 (Strongly Disagree) to 5 (Strongly Agree), with higher scores indicating a stronger tendency toward social dependence.

The fifth section of the survey employed the 6-item Exercise Addiction Inventory (EAI-6) developed by Terry et al. (2004) to assess participants’ compulsive exercise behaviors and psychological dependence on physical activity. Sample items include: “Exercise is the most important thing in my life” and “If I have to miss an exercise session, I feel moody and irritable.” Each item was rated on a five-point Likert scale ranging from 1 (Strongly Disagree) to 5 (Strongly Agree), with higher scores indicating a greater tendency toward exercise addiction.

### 3.3 Data analysis

In this study, structural equation modeling (SEM) combined with partial least squares SEM (PLS-SEM) was employed to examine the proposed model. SEM is a widely recognized approach for analyzing latent variables and testing relationships among constructs within measurement and structural models (Hair et al., 2012). Following the two-step procedure recommended by Anderson and Gerbing (1988), we first assessed the measurement model to evaluate the reliability and validity of the instruments, with the lowest Cronbach’s alpha recorded at 0.768, indicating excellent internal consistency. Subsequently, the structural model was analyzed using maximum likelihood estimation to examine the associations among mindfulness, cognitive reappraisal, perfectionism, and work addiction. Additionally, 5,000 bootstrap samples were used to test the indirect effects between high-intensity emotional intelligence and psychological detachment. Finally, model performance was evaluated by examining fit indices and path coefficients for the hypothesized model.

## 4 Results

### 4.1 Measurement model

The reliability and validity assessment of latent variables incorporated confirmatory factor analysis (CFA) through SmartPLS. All variables demonstrated Cronbach’s *α* values surpassing 0.8 (refer to Table 2), affirming robust internal consistency within the model structure as guided by Fornell and Larcker (1981). Additionally, the average variance extraction (AVE) for each variable exceeded 0.6 (as noted in Table 2), surpassing the minimal acceptable threshold of 0.5. Furthermore, the composite reliability (CR) of each latent variable surpassed 0.8, underscoring the model’s robust convergent validity. The resilience of convergent validity across proposed models was well-established. Factor loadings from principal component factor analysis ranged from 0.796 to 0.942 (refer to Table 2), reinforcing the measurement model’s robust construct validity.

**Table 2:** Reliability and validity.

Items	Factor loadings	Cronbach’s α	CR	AVE
Difficulties in Emotion Regulation (DER)		0.970	0.974	0.825
DER1	0.922			
DER2	0.906			
DER3	0.905			
DER4	0.908			
DER5	0.913			
DER6	0.883			
DER7	0.920			
DER8	0.907			
State Anxiety (SA)		0.927	0.976	0.663
SA1	0.904			
SA2	0.915			
SA3	0.962			
SA4	0.937			
SA5	0.941			
SA6	0.942			
Social Dependence (SD)		0.893	0.919	0.656
Emotional Dependency (ED)		0.866	0.918	
ED1	0.926			
ED2	0.831			
ED3	0.906			
Functional Dependency (FD)		0.768	0.866	
FD1	0.873			
FD2	0.796			
FD3	0.809			
Exercise Addiction (EA)		0.941	0.953	0.773
EA1	0.848			
EA2	0.901			
EA3	0.883			
EA4	0.890			
EA5	0.873			
EA6	0.882			

Cα, Cronbach’s alpha; AVE, Average Variance Extracted; CR, Composite Reliability.

**Figure 2:**
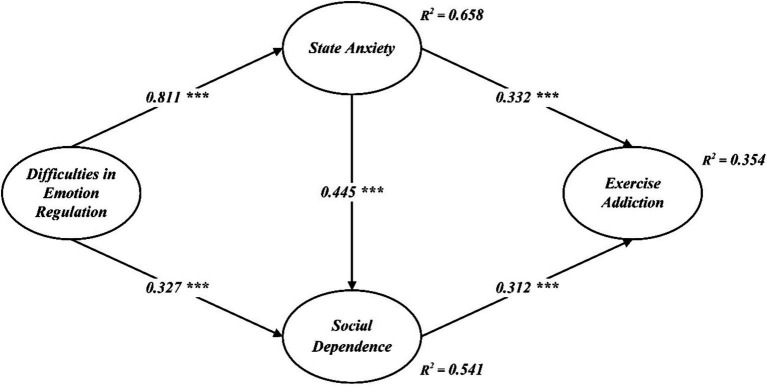
Structural path analysis model.

According to Fornell and Larcker (1981), diagonal values of the square root of AVE are bigger than inter-item correlation values, demonstrates discriminant validity. Item loadings, alpha, AVE and CR values are shown in Table 2, while discriminant validity scores are shown in Table 3. The outcomes revealed that the model is appropriate for the structural model evaluation employed in the final analysis (Figure 2).

**Table 3:** Discriminant validity.

Constructs	DER	EA	SA	SD
DER	0.908			
EA	0.606	0.879		
SA	0.811	0.553	0.934	
SD	0.688	0.547	0.71	0.81

DER, Difficulties in Emotion Regulation; EA, Exercise Addiction; SA, State Anxiety; SD, Social Dependence.

### 4.2 Structural model

The study evaluated the model fit using the standardized root mean square residual (SRMR). The SRMR value is a standardized residuals index determined by comparing the observed covariance with the hypothesized matrix. A range of SRMR values of 0.1 or lower is considered acceptable (Hu and Bentler, 1998). Based on the results, the estimated SRMR value was 0.082, which is considered a satisfactory model fit.

The structural path model in Table 4 and Figure 1 shows that the significant positive correlation between difficulties in emotion regulation and state anxiety (*β* = 0.811, *t* = 13.395, *p* < 0.001), supporting H1; and there was a significant positive correlation between difficulties in emotion regulation and social dependence was (*β* = 0.327, *t* = 4.181, *p* < 0.001), supporting H2; and there was a significant positive correlation between state anxiety and social dependence (*β* = 0.445, *t* = 5.760, *p* < 0.001), supporting H3; and there was a significant positive correlation between social dependence and exercise addiction (*β* = 0.332, *t* = 6.340, *p* < 0.001), supporting H4; there was a significant positive correlation between state anxiety and exercise addiction (*β* = 0.312, *t* = 5.359, *p* < 0.001), supporting H5.

**Table 4:** Path coefficients.

No.	Path	β	Mean	T statistics	*p* values	LLIC	ULIC
H1	DER→SA	0.811	0.812	25.478	0.000	0.745	0.869
H2	DER→SD	0.327	0.326	4.181	0.000	0.174	0.481
H3	SA→SD	0.445	0.446	5.760	0.000	0.293	0.596
H4	SA→EA	0.332	0.331	6.340	0.000	0.229	0.435
H5	SD→EA	0.312	0.313	5.359	0.000	0.199	0.425

DER, Difficulties in Emotion Regulation; EA, Exercise Addiction; SA, State Anxiety; SD, Social Dependence.

The model’s explanatory power was evaluated by assessing the goodness of fit, determined through the strength of each structural path indicated by the R^2^ value for the dependent variable (Shmueli and Koppius, 2011). According to Falk and Miller (1992), an R^2^ value equal to or greater than 0.1 signifies a good fit. As shown in Table 5, all R^2^ values exceeded 0.1, confirming the model’s predictive capability. Additionally, Q^2^ was used to ascertain the predictive relevance of the endogenous constructs; a Q^2^ value above zero indicates predictive relevance. The results in Table 5 demonstrate significant predictive power for the constructs.

**Table 5:** Outcomes of R^2^ and Q^2^ values.

Endogenous constructs	R^2^	Adjusted R^2^	Q^2^
EA	0.354	0.351	0.386
SD	0.541	0.539	0.219
SA	0.658	0.657	0.193

EA, Exercise Addiction; SA, State Anxiety; SD, Social Dependence.

The researchers proposed that difficulties in emotion regulation impacts exercise addiction through two mediating factors: state anxiety and social dependence. To test these mediation effects, bootstrapping methods were employed (Bollen and Stine, 1990). As presented in Table 6, both cognitive fusion and perfectionism had a positive effect on the relationship between mindfulness and stress self-management (standard indirect effect = 0.076, *p* < 0.001), thereby supporting H6.

**Table 6:** Mediation analysis.

No	Path	Original sample (O)	Sample mean (M)	Standard deviation (STDEV)	T statistics (|O/STDEV|)	*p* values
H6	Total indirect effect of DER→EA	0.484	0.485	0.030	15.974	0.000
	Total effect of DER→EA	0.484	0.485	0.030	15.974	0.000

DER, Difficulties in Emotion Regulation; EA, Exercise Addiction.

## 5 Discussion

### 5.1 Theoretical contributions

This study offers several theoretical contributions to the emerging literature on exercise addiction, particularly within the context of aging populations. First, by focusing on older adults, this research expands the scope of exercise addiction studies, which have traditionally concentrated on younger, fitness-oriented individuals (Hausenblas and Downs, 2002; Egorov and Szabo, 2013). More importantly, this study extends existing theoretical models by empirically testing an integrated mediation framework that simultaneously incorporates affective (difficulties in emotion regulation) and social (social dependence) pathways linking exercise behavior to exercise addiction in a post-retirement population. In doing so, it highlights the need to re-examine behavioral health risks in nontraditional populations, such as retirees, whose vulnerability to psychological dependence on exercise may stem from distinct socioemotional challenges related to aging and role transition (Bordia et al., 2020; Goll et al., 2015).

Second, this study advances theoretical understanding by testing and supporting an integrated psychological pathway model linking difficulties in emotion regulation, state anxiety, and social dependence to exercise addiction among older adults. Previous studies on exercise addiction have predominantly focused on describing behavioral symptoms or physiological consequences of excessive exercise (Lichtenstein et al., 2017; Freimuth et al., 2011), with comparatively limited attention to the emotional and interpersonal processes underlying such behaviors in later life. By simultaneously incorporating affective regulation processes and relational needs within a single analytical framework, the present study extends prior work and offers a more comprehensive account of the psychological mechanisms through which compulsive exercise may develop in post-retirement populations.

Third, the findings highlight the dual mediating roles of state anxiety and social dependence, thereby adding theoretical nuance to existing models of behavioral addiction. Specifically, the results suggest that difficulties in emotion regulation may give rise to maladaptive exercise behaviors through both internal emotional pathways (heightened anxiety) and external social pathways (increased reliance on exercise for social connection and validation). This distinction refines current understandings of exercise addiction by demonstrating that psychological dependence on exercise among older adults cannot be explained solely by affective dysregulation. Rather, it also reflects unmet social identity needs and interpersonal validation processes that become particularly salient during the post-retirement transition.

Finally, the research responds to the broader call for more gerontology-informed perspectives in health psychology. It challenges the normative assumption that more exercise is always better and draws attention to the “dark side” of health-promoting behaviors when taken to extremes. In this regard, the findings resonate with broader critiques of healthism and overmedicalization in later life, where responsibility for health is increasingly individualized and intensive self-regulation of the body is socially encouraged. By doing so, the study lays a theoretical foundation for future work aiming to establish age-appropriate frameworks for recognizing, measuring, and intervening in exercise addiction in aging populations.

### 5.2 Practical implications

The findings of this study offer important practical implications for mental health promotion, behavioral intervention, and social support system design targeting older adults.

First, the identification of emotion regulation difficulties and state anxiety as key psychological drivers of exercise addiction highlights the need to incorporate emotional well-being monitoring into community-based exercise programs for older adults. Current exercise initiatives often focus solely on physical health outcomes, neglecting the psychological motivations and vulnerabilities that may underlie excessive participation. By integrating regular psychological screening tools and offering emotional regulation training (e.g., mindfulness workshops, cognitive reappraisal techniques), practitioners can help reduce the emotional triggers that may escalate into compulsive exercise behaviors.

Second, the mediating role of social dependence underscores the importance of developing diversified social engagement opportunities for retirees. As exercise in older adults is not merely a health behavior but also a tool for emotional connection and social identity, community centers, health organizations, and local governments should consider providing broader, non-exercise-based avenues for social inclusion—such as group discussions, intergenerational programs, or structured volunteering. These alternatives may help fulfill older adults’ need for belonging and interpersonal validation without over-relying on physical activity.

Third, the results call attention to the need for education and awareness regarding the potential risks of excessive exercise, especially within families and community networks that may unintentionally reinforce addictive tendencies through praise or misinterpretation. Public health messaging should aim to balance the promotion of physical activity with recognition of its potential psychological misuse. Campaigns should target not only older adults themselves, but also caregivers, exercise instructors, and family members who play a role in shaping exercise habits.

Finally, the proposed pathway model provides a theoretical basis for early risk detection and personalized intervention. Mental health professionals and community healthcare providers could use screening tools based on the variables identified in this study—such as emotion regulation difficulty, anxiety symptoms, and social dependence—to assess the likelihood of problematic exercise behaviors. Tailored support plans could then be implemented, ranging from psychological counseling to exercise moderation planning.

### 5.3 Limitations

Despite offering novel insights into the psychological mechanisms underlying exercise addiction among older adults, this study has several limitations that warrant consideration. First, the cross-sectional design limits the ability to infer causal relationships between difficulties in emotion regulation, state anxiety, social dependence, and exercise addiction. Longitudinal or experimental designs are needed to verify the temporal ordering and directionality of these associations. Second, all data were collected via self-report questionnaires, which may be subject to social desirability bias and common method variance. Future studies could incorporate objective indicators of exercise behavior and physiological regulation, such as accelerometer-based activity data or heart rate variability, to complement self-report measures and improve measurement validity. Third, the sample was drawn from community-dwelling older adults in specific regions of China, which may restrict the generalizability of findings to other demographic groups or cultural contexts. Further cross-cultural or multi-site studies could clarify the broader applicability of the proposed model. Lastly, while this study focused on three key psychological variables, other potentially relevant factors, such as personality traits, cognitive styles, and social norms, were not examined. Personality traits including perfectionism and neuroticism are theoretically linked to rigid behavioral patterns, heightened emotional reactivity, and vulnerability to psychological dependence. Expanding the model to include these variables may provide a more comprehensive understanding of exercise addiction among older adults.

## 6 Conclusion

As the aging population continues to grow, understanding the mental health challenges faced by post-retirement individuals has become increasingly urgent. This study contributes to the literature by testing and supporting a pathway model in which difficulties in emotion regulation are associated with exercise addiction via the mediating roles of state anxiety and social dependence. The findings highlight that while physical activity is widely regarded as beneficial, excessive and compulsive exercise behaviors among older adults may stem from deeper psychological vulnerabilities, such as affective dysregulation and unmet emotional or social needs. These results not only enrich our theoretical understanding of non-substance addiction in late adulthood but also highlight the importance of balanced exercise interventions that integrate emotion regulation training, social support, and mental health awareness. By clarifying the psychological drivers of exercise addiction, this study offers a foundation for more targeted and effective strategies in aging health promotion and geriatric mental healthcare.

## Data Availability

The raw data supporting the conclusions of this article will be made available by the authors, without undue reservation.
